# A Rare Case: Acute Liver Injury Uncovering a Diagnosis of B-cell Acute Lymphoblastic Leukemia in a Young Adult

**DOI:** 10.7759/cureus.35274

**Published:** 2023-02-21

**Authors:** Emily Venvertloh, David Karp, Henrik H Ghantarchyan, Kambiz Raoufi

**Affiliations:** 1 Internal Medicine, Arrowhead Regional Medical Center, Colton, USA

**Keywords:** hematology, acute hepatitis, bone marrow, acute liver failure, b-cell all

## Abstract

Acute liver injury can be seen in a myriad of disease states such as chronic alcoholism, hepatitis, and hepatocellular carcinoma. When considering acute liver injury in a young adult, the list of differential diagnoses is much more narrow. One rare cause can be acute lymphoblastic leukemia (ALL), a hematologic malignancy that can be seen in the young adult population. We present a rare case of an 18-year-old male with no prior medical history who presented with abdominal pain and nausea. A complete workup for acute liver injury aided us in uncovering a diagnosis of B-cell ALL. This case highlights the importance of a broad differential in acute liver injury and consideration of ALL as a cause of acute liver injury, especially in young adults.

## Introduction

Acute lymphoblastic leukemia (ALL), an aggressive hematologic malignancy, is characterized by the overproduction of immature white blood cells, mainly lymphoblasts, in the blood and bone marrow. In young adults, ALL is a relatively rare malignancy affecting approximately 6,500 individuals in the United States each year [1]. Individuals with ALL often present with fatigue and pallor caused by anemia, bruising or bleeding caused by thrombocytopenia, and infections as a result of neutropenia [2]. Our case highlights an individual who presents with nausea, vomiting, and abdominal pain in the setting of new-onset acute liver injury. Although infiltration of the liver by hematologic malignancies is an uncommon cause of liver injury, Heincelman et al. reported a similar case of an 18-year-old male with ALL who initially presented with hepatitis and acute kidney injury [3]. Because there are limited reports of acute liver injury as the initial findings of ALL, our case is significant in its atypical presentation of an underlying hematologic malignancy in a young adult patient.

## Case presentation

An 18-year-old Middle Eastern male with no known prior medical history presented to local urgent care with two weeks of intermittent abdominal pain and generalized weakness a day before arrival at our hospital. He denied any alterations in his regular diet or any drug, alcohol, or tobacco use. He had one tattoo performed by a licensed tattoo artist. He reported a recent trip to Lebanon a month prior where he ingested undercooked meat and unfiltered water. There were no associated night sweats, fever, chills, unexpected weight loss, hematemesis, melena, hematochezia, and diarrhea. The patient’s only known pertinent family history was leukemia in his paternal uncle.

**Figure 1:**
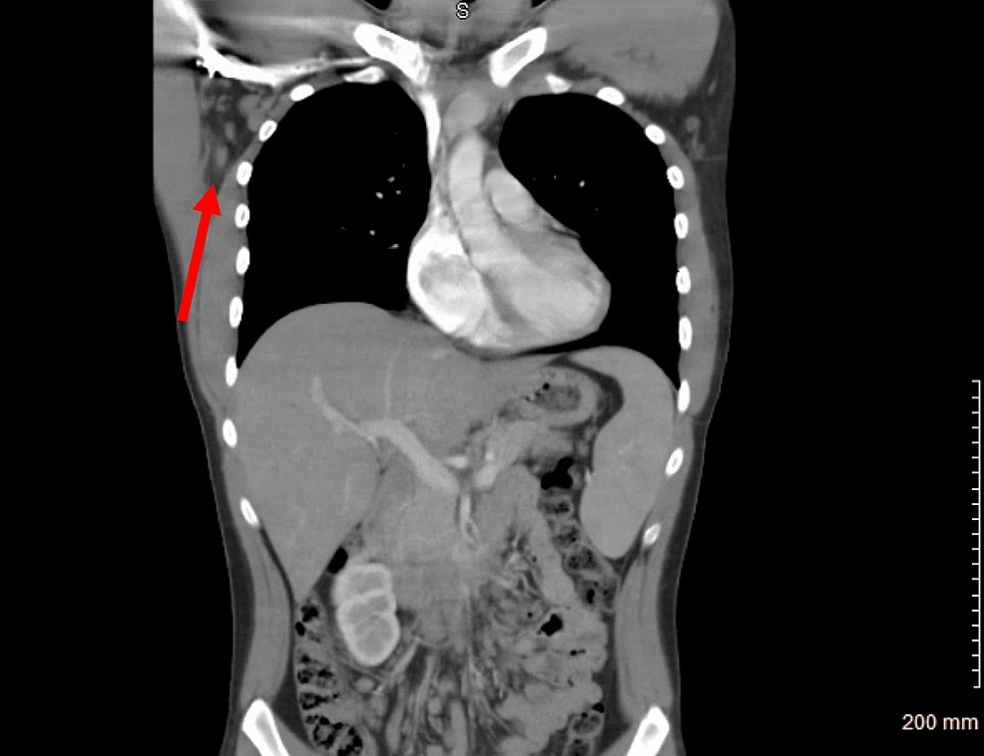
Computed tomography (CT) abdomen and pelvis with intravenous contrast with the arrow signifying axillary lymphadenopathy

On arrival at the emergency department, his vital signs showed tachycardia with a rate of 114/min. On physical exam, bilateral axillary lymphadenopathy was noted. Initial labs were pertinent for anemia, thrombocytopenia, and increased blast cells (Table 1). There was also transaminitis and direct hyperbilirubinemia (Table 2). Initial coagulation tests showed activated partial thromboplastin time (aPTT) 36 (25.4-36.8 seconds), prolonged prothrombin time of 16 seconds (11.8-14.2 seconds), and international normalized ratio (INR) 1.35 (<1.10). Iron studies showed iron 214 ug/dl (50-160 ug/dl), ferritin 1,846 ng/ml (20-300 ng/ml), and total iron-binding capacity (TIBC) 238 ug/dl (250-450 ug/dl). A computed tomography (CT) of the abdomen and pelvis showed prominent bilateral axillary lymph nodes with no splenomegaly or hepatomegaly (Figure 1). An ultrasound of the abdomen showed a normal flow of the hepatic portal vein without hepatomegaly or hepatic masses.

**Table 1:** Significant laboratory values in complete blood count (CBC) Legend: μL = microliter, ug = microgram, dL = deciliter, U/L = units per liter, g = gram, mg = milligram

Blood test results (units)	Patient value	Reference range
WBC, x10^3^/uL	4.6	4.5-11.1
Hemoglobin (g/dL)	11.2	13.0-17.0
MCV (fL)	83	80.0-100.0
Platelets, 10^3^/uL	94	120-360
Blasts %	12	<1
Lymphocytes %	66	%
Monocytes %	5	%

**Table 2:** Elevation in liver function tests (LFTs) μL = microliter, ug = microgram, dL = deciliter, U/L = units per liter, g = gram, mg = milligram, alanine transaminase (ALT), aspartate transaminase (AST), gamma-glutamyl transferase (GGT), lactate dehydrogenase (LDH)

Blood test results (units)	Patient value	Reference range
Alkaline phosphatase (IU/L)	155	34-104
Albumin (g/dL)	4.5	3.5-4.9
Total protein (g/dL)	6.6	6.0-8.0
Uric acid (mg/dL)	7.9	2.6-7.2
Lipase (U/L)	25	10-60
AST (U/L)	856	5-40
ALT (U/L)	1,434	5-40
Bilirubin, direct (mg/dL)	1.0	0-0.4
Total bilirubin (mg/dL)	1.8	0.0-1.2
GGT (U/L)	135	5-37
LDH (U/L)	858	120-230

**Figure 2:**
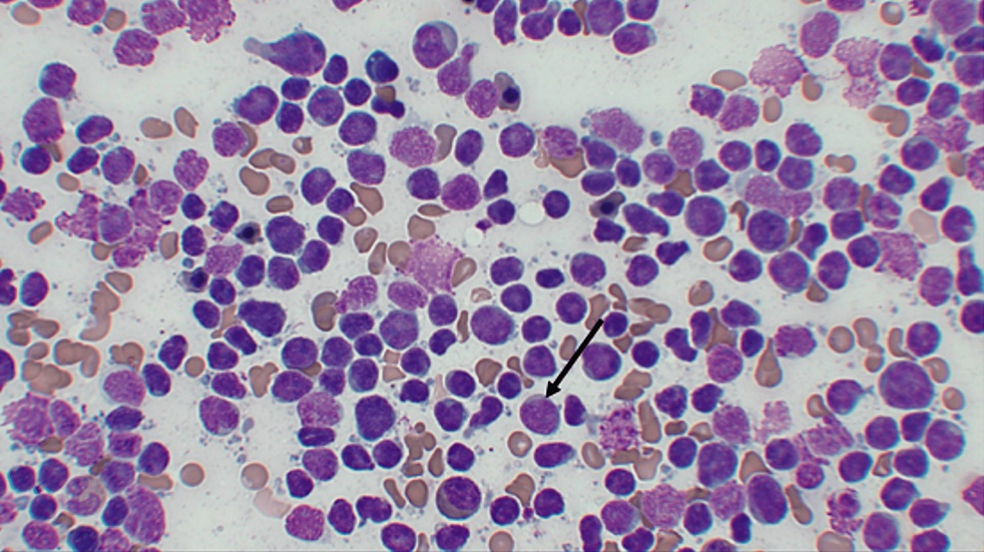
Bone marrow biopsy A low-powered section of the core needle biopsy shows the proliferation of immature blasts (black arrow) as the dominant cell type (80%) bone marrow cellular elements with minimal cytoplasm, immature chromatin pattern, high nucleus-to-cytoplasm ratio, and prominent nucleoli indicating lymphoid lineage.

Further workup included a peripheral blood smear, which was concerning for myeloblasts. As suspicion for leukemia increased, flow cytometry revealed CD45-dim population positive for CD19, CD22, CD10, and TdT without significant CD34, surface light chains, or other lymphoid and myeloid cells. There were 49% blasts, 7% granulocytic cells, 42% lymphocytoid cells, and 1% monocytoid cells. Given elevated blasts on peripheral smear and flow cytometry, a bone marrow biopsy was completed. The bone marrow biopsy revealed greater than 90% cellularity with diminished trilineage hematopoiesis, which can be seen in Figure 2. There were no abnormalities by fluorescence in situ hybridization (FISH) or cytogenetics noted.

In the following days, liver enzymes reached a peak of aspartate transaminase (AST) 1,628 U/L and alanine transaminase (ALT) 3,106 U/L, which eventually steadily decreased with time while direct bilirubin continued to increase to 9.7 mg/dL (0.0 - 1.2 mg/dL). During the hospital stay, the patient was shown to have worsening jaundice and scleral icterus but with no alteration in mentation. As the patient had a steady rise in his liver function tests, a liver biopsy was performed and showed severe portal and periportal chronic inflammation with multicellular necrosis. Further assessment of other causes of liver injury was unremarkable, including the hepatitis panel checking for hepatitis A, hepatitis B, hepatitis C, and hepatitis E was negative. Testing was also negative for other viruses, including cytomegalovirus and Epstein-Barr virus. Autoimmune causes were also considered, including antinuclear antibodies (ANA), antineutrophil cytoplasmic antibodies (ANCA), proteinase-3 antibodies, mitochondrial antibodies, anti-smooth muscle antibodies, liver-kidney microsomal antibodies, and immunoglobulin G (IgG) subclasses, which were all negative. A urine toxicology screen, acetaminophen level, ethanol level, and salicylate level were also negative.

The decision was made to transfer the patient to a higher level of care at an outside hospital (OSH) for the treatment of ALL. At the OSH, he was started on prednisone 30 mg/m^2^/dose twice daily for 10 days, followed by prednisone 60 mg twice daily for 21 days. Prior to starting chemotherapy, liver enzymes and bilirubin levels had decreased to ALT 485 (7-45 U/L), AST 48 (0-35 U/L), and total bilirubin 2.9 (0.0-1.0 mg/dL). The induction chemotherapy regimen included pegaspargase 2,500 U/m^2^ IV (intravenous), vincristine 2 mg IV, daunorubicin 52 mg IV, cytarabine 60 mg intrathecal (IT), and methotrexate 15 mg IT. Micafungin 100 mg IV daily was started as prophylaxis for 30 days. The patient was also started on ursodiol 300 mg orally two times daily for hyperbilirubinemia and allopurinol 100 mg/m^2^ three times daily for tumor lysis.

## Discussion

This article explores a case of acute liver injury as the presenting symptom of ALL in a young adult. ALL is the second most common acute leukemia in adults and the most common pediatric malignancy, with 80% of ALL cases occurring in pediatric patients [1,4]. ALL is more common in pediatric patients but has a higher mortality rate in adults [4]. Neoplastic replication of stem cell progenitors of bone marrow and lymphoid organs leads to the development of ALL [5]. Common and, at times, non-specific symptoms include B symptoms - fever, unexpected weight loss, night sweats, and fatigue. Lymphadenopathy, splenomegaly, and hepatomegaly can occur due to extramedullary involvement in 20% of patients [1]. However, acute liver failure is a rarer occurrence and has been reported in previous case reports, mainly in the pediatric population [6,7] and less commonly in the adult population [3]. Segal et al. conducted a retrospective study over a five-year period in pediatric patients who were diagnosed with ALL and found to have abnormal liver enzymes. The study of 147 patients found one-third of the participants to have abnormal AST and/or ALT values and 3.4% with conjugated hyperbilirubinemia [8]. There is more literature on the pediatric population regarding ALL, and our case highlights the importance of considering the young adult and adult populations, as these two populations have increased mortality rates and adults have inferior long-term prognoses as compared to children [9].

A possible mechanism for the cause of an acute rise in transaminases in the setting of leukemia has been hypothesized in current literature. Liver injury can be due to secondary causes, including autoimmune hepatitis, viral infection, sepsis, or leukemic infiltration of the liver leading to hypoxia and ischemia [6,10]. Aspartate transaminase (AST) typically rises first and then with ongoing damage, ALT can surpass AST with prolonged hepatocellular injury [5]. This pattern was reflected in our case with ALT nearly double AST at the peak. At the time of diagnosis, liver injury is often not present on laboratory tests but postmortem studies demonstrated >95% of cases of ALL were shown to have hepatic infiltration [10].

In evaluating a previously healthy young adult male with an acute rise in liver enzymes, it is essential to start with a broad differential. The differential can include but is not limited to viral, drug-induced, autoimmune, acute ingestion, malignancy, hypoxia-induced, Wilson disease, Budd-Chiari, and sepsis [11]. The two leading causes are hepatitis (hepatitis A, C, and E) and drug-induced, specifically acetaminophen [11]. For this case, the main considerations were infectious from hepatitis or other viral illness, given recent travel to Lebanon. Other considerations included toxic ingestion of acetaminophen or other sources, autoimmune, and malignancy, given elevated blasts on differential and lymphadenopathy. In the infectious work-up, special attention was paid to hepatitis, syphilis, Cytomegalovirus, Epstein-Barr virus, herpes simplex virus, and human immunodeficiency virus, which were all negative. Autoimmune etiology was considered and included autoimmune hepatitis, primary biliary cholangitis, and primary sclerosing cholangitis. The autoimmune workup showed non-elevated antinuclear antibodies (ANA), ANCA, proteinase-3 antibody, mitochondrial antibody, actin smooth muscle antibody, liver-kidney microsomal antibody, and immunoglobulin G panel. There was also an initial concern for acetaminophen or salicylate overdose, substance abuse, and alcohol use, but all relevant lab results were negative. As the case progressed, it became more apparent that the patient’s presentation was likely due to leukemia. The initial findings that made malignancy more apparent were axillary lymph nodes on CT of the abdomen and pelvis and elevated blast cells on complete blood count (CBC) and peripheral smear. Other causes of the acute liver injury in addition to malignancy were considered, but this was ruled out with an extensive workup as stated above. The diagnosis of ALL was ultimately confirmed with a bone marrow biopsy.

Treatment for ALL can vary depending on the patient's age, leukocyte count at the time of diagnosis, treatment response, and more specialized factors, including specific translocations and aberrations identified on analysis [4]. As part of the conventional protocol, treatment of ALL begins with chemotherapy induction using a combination of anthracyclines, vincristine, asparaginase, intrathecal methotrexate, mercaptopurine, cyclophosphamide, and steroids. This is followed by consolidation therapy and/or bone marrow transplantation and then maintenance therapy [4].

Acute liver injury as the presenting symptom of ALL can create complications in treatment planning. Many of the induction chemotherapy agents, including vincristine and anthracyclines, are hepatically metabolized. If there is already dysfunction present, this can further increase the risk of toxicity in these patients [6]. This could lead to a possible delay in treatment or the need for dose reduction in the chemotherapeutic agents utilized due to concern for further hepatotoxicity. Asparaginase, one of the newer chemotherapeutic agents used against ALL, works by depleting asparagine. This derivative of *Escherichia coli (E. coli)* has shown promising results, with remission rates of 93%. However, it is known to cause hepatotoxicity, which can be a limiting factor in its use. Known risk factors for developing toxicity with asparaginase use are seen in individuals who are obese, of Hispanic ethnicity, or with a known SOD2 genetic mutation. The rate of hepatotoxicity is variable, with up to 60% of patients experiencing transaminitis [12]. There have been studies that showed that the addition of steroids can decrease the liver enzymes to a level where initiation of chemotherapy would have decreased the risk of toxicity [7]. 

## Conclusions

Acute lymphoblastic leukemia is a hematologic disorder more common in children than adults, which often presents with non-specific B-type symptoms and in some cases, hepatomegaly, splenomegaly, or lymphadenopathy. This patient who was diagnosed with B-cell acute lymphoblastic leukemia had an atypical presentation with acute liver injury. This case demonstrates that acute lymphoblastic leukemia can cause acute hepatitis, and this can be the initial presentation of leukemia in a young adult. A bone marrow biopsy can help identify this disease entity once other, more common etiologies of hepatitis have been ruled out.
